# Dose-Response of Paraxanthine on Cognitive Function: A Double Blind, Placebo Controlled, Crossover Trial

**DOI:** 10.3390/nu13124478

**Published:** 2021-12-15

**Authors:** Dante Xing, Choongsung Yoo, Drew Gonzalez, Victoria Jenkins, Kay Nottingham, Broderick Dickerson, Megan Leonard, Joungbo Ko, Mark Faries, Wesley Kephart, Martin Purpura, Ralf Jäger, Shawn D. Wells, Ryan Sowinski, Christopher J. Rasmussen, Richard B. Kreider

**Affiliations:** 1 Exercise & Sport Nutrition Lab, Human Clinical Research Facility, Department of Health & Kinesiology, Texas A&M University, College Station, TX 77843, USA; dantexing@tamu.edu (D.X.); choongsungyoo@tamu.edu (C.Y.); dg18@tamu.edu (D.G.); victoria.jenkins@tamu.edu (V.J.); kvnottingham@tamu.edu (K.N.); dickersobl5@email.tamu.edu (B.D.); meganleonard10@tamu.edu (M.L.); joungboko10@tamu.edu (J.K.); Mark.Faries@ag.tamu.edu (M.F.); ryansowinski6@gmail.com (R.S.); crasmussen@tamu.edu (C.J.R.); 2 Texas A&M AgriLife Extension, Texas A&M University, College Station, TX 77843, USA; 3 Department of Kinesiology, University of Wisconsin, Whitewater, WI 53190, USA; kephartw@uww.edu; 4 Increnovo LLC, Milwaukee, WI 53202, USA; martin.purpura@increnovo.com (M.P.); ralf.jaeger@increnovo.com (R.J.); shawn@ingeniousingredients.com (S.D.W.); 5 Ingenious Ingredients L.P., Lewisville, TX 75056, USA

**Keywords:** mental performance, nootropics, caffeine alternative, paraxanthine

## Abstract

Paraxanthine (PXN) is a metabolite of caffeine that has recently been reported to enhance cognition at a dose of 200 mg. Objective: To determine the acute and short-term (7-day) effects of varying doses of PXN on cognitive function and side effects. Methods: In a double blind, placebo-controlled, crossover, and counterbalanced manner, 12 healthy male and female volunteers (22.7 ± 4 years, 165 ± 7 cm, 66.5 ± 11 kg, 24.4 ± 3 kg/m^2^) ingested 200 mg of a placebo (PLA), 50 mg of PXN (ENFINITY™, Ingenious Ingredients, L.P.) + 150 mg PLA, 100 mg PXN + 100 mg PLA, or 200 mg of PXN. With each treatment experiment, participants completed side effect questionnaires and donated a fasting blood sample. Participants then performed a series of tests assessing cognition, executive function, memory, and reaction time. Participants then ingested one capsule of PLA or PXN treatments. Participants then completed side effects and cognitive function tests after 1, 2, 3, 4, 5, and 6 h of treatment ingestion. Participants continued ingesting one dose of the assigned treatment daily for 6-days and returned to the lab on day 7 to donate a fasting blood sample, assess side effects, and perform cognitive function tests. Participants repeated the experiment while ingesting remaining treatments in a counterbalanced manner after at least a 7-day washout period until all treatments were assessed. Results: The Sternberg Task Test (STT) 4-Letter Length Present Reaction Time tended to differ among groups (*p* = 0.06). Assessment of mean changes from baseline with 95% CI’s revealed several significant differences among treatments in Berg-Wisconsin Card Sorting Correct Responses, Preservative Errors (PEBL), and Preservative Errors (PAR Rules). There was also evidence of significant differences among treatments in the Go/No-Go Task tests in Mean Accuracy as well as several time points of increasing complexity among STT variables. Finally, there was evidence from Psychomotor Vigilance Task Test assessment that response time improved over the series of 20 trials assessed as well as during the 6-h experiment in the PXN treatment. Acute and short-term benefits compared to PLA were seen with each dose studied but more consistent effects appeared to be at 100 mg and 200 mg doses. No significant differences were observed among treatments in clinical chemistry panels or the frequency or severity of reported side effects. Results provide evidence that acute ingestion of 100 mg and 200 mg of PXN may affect some measures of cognition, memory, reasoning, and response time as well as help sustain attention. Additionally, that acute and daily ingestion of PXN for 7 days is not associated with any clinically significant side effects. Conclusions: PXN may serve as an effective nootropic agent at doses as low as 50 mg.

## 1. Introduction

Caffeine (CA) is the most popular stimulant for increasing focus, alertness, concentration as well as promote cognitive health benefits [1,2,3]. The pharmacokinetics, ergogenic value, and effects of CA on weight loss have been well-documented [2,4,5,6,7,8]. However, genetics can affect an individual’s response to caffeine ingestion [9]. Variants in a gene called CYP1A2, which encodes the enzyme cytochrome P450, which is responsible for >95% of caffeine metabolism determine the speed of CA clearance. Less than half of the population carry the CYP1A2 gene associated with fast metabolism. Some evidence suggest that higher CA intakes increases the risk of insulin resistance [10], high blood pressure [11], and heart attacks [12] in intermediate and slow but not rapid caffeine metabolizers [8] with fast metabolizer seeing greater improvements in athletic performance [13,14] and reduced appetite [15,16].

Paraxanthine (1,7-dimethylxanthine, PXN) is the primary metabolite of CA in humans [17]. PXN has a shorter half-live and greater plasma clearance in comparison to CA as well as the other metabolites of caffeine (i.e., theobromine or 3,7-dimethylxanthine [TB] and theophylline or 1,3-dimethylxanthine [TP]) [4]. Studies indicate that PXN has less toxicity [18] and anxiety promoting effects than CA [19]. Moreover, TP has been reported to promote tachycardia, arrhythmias, nausea, and diarrheas [20]. By eliminating metabolism into TB and TP and avoiding the genetic differences in CA metabolism, PXN may not only serve as a safer stimulant compared to CA, but preliminary studies, primarily in animals, indicate that PXN may be more effective than CA in enhancing cognition [21,22,23,24].

We recently reported that one-time ingestion of 200 mg of PXN improved response time to cognitive challenges, as well as measures of memory, reasoning, and attention over a six-hour period in healthy adults [25]. Theoretically, PXN may provide an alternative to CA, particularly in individuals who are genetically less sensitive to CA and/or experience untoward side effects. However, more research is needed to determine dose response relationships, as well as safety of short-term daily use. The objectives of this study were: (1) to determine the acute minimal and optimal dose of acute PXN ingestion on measures of cognition, memory, vigilance, and side effects; (2) to determine whether 7-days of PXN ingestion provides sustained and/or additional effects on cognitive function; and, (3) to examine the safety of 7-days of PXN supplementation on subjective ratings of side effects and clinical chemistry panels. We hypothesized that PXN would significantly affect primary outcome measures of cognitive and executive function in a dose responsive manner with no significant effects on secondary outcomes related to safety.

## 2. Methods

### 2.1. Experimental Design

This study was performed in a university-setting as a double blind, placebo-controlled, crossover, and counterbalanced clinical trial. The study was approved by the Human Protection Program Institutional Review Board (IRB2019-00807F) in compliance with the Declaration of Helsinki standards for ethical conduct of human participant research. This study was also registered with the International Standard Randomized Control Number (ISRCTN) registry (ISRCTN68592648). The independent variable was stimulant ingestion. The primary outcomes were measures of cognitive function. Secondary outcomes included changes in clinical blood chemistry and subjective ratings of symptoms and side effects after acute and one week of treatment.

### 2.2. Participants

**Figure 1:**
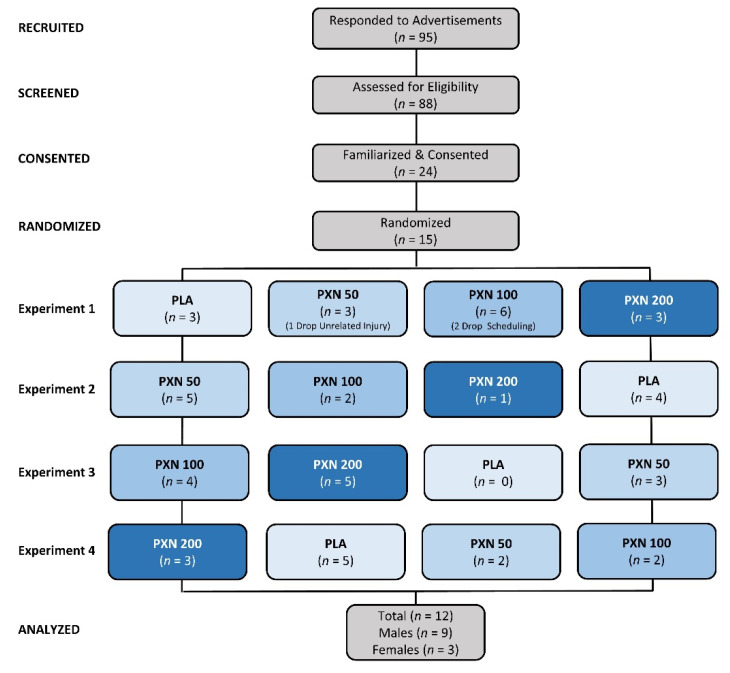
Consolidated Standards of Reporting Trials (CONSORT) diagram for the placebo (PLA) and paraxanthine (PXN) treatments.

Participants were recruited from the faculty, staff and student population, as well as the local community. Individuals expressing interest in participating in the study underwent a phone screening to determine general eligibility. Participants were eligible to participate in the study if they: (1) were healthy males and females between the ages of 18–59 years; (2) had no history of cognitive dysfunction; and, (3) were willing to give voluntary, written, and informed consent to participate in the study. Participants were not eligible to participate if they: (1) had a known cognitive deficit condition; (2) had a known allergy to wheat flour; (3) had a sleep disorder; (4) had metabolic, cardiovascular or pulmonary disease; (5) had hypertension, cardiac arrhythmias, migraine headaches, or anxiety; (6) had gastrointestinal reflux disease or ulcers; (7) were pregnant or breastfeeding; (8) were currently taking prescription medications that may affect study outcomes within the prior month; and/or, (9) had been advised by their physician to abstain or restrict from caffeine and/or other stimulants. Those meeting phone screening entry criteria were invited to attend a familiarization session to sign consent forms and confirm eligibility to participate in the study. Figure 1 presents a Consolidated Standards of Reporting Trials (CONSORT) diagram. A total of 95 individuals responded to study advertisements, and 88 underwent phone screening to assess eligibility. Of these, 24 individuals were familiarized and consented to participate in the study. Eight participants decided not to participate before testing began due to scheduling conflicts. Fifteen participants were randomized and allocated into treatments as shown. Three participants withdrew after the first experiment due to time constraints (2) and an unrelated injury (1). A total of 12 participants completed the study (3 females, 9 males). Participants were healthy males and females (22.8 ± 4 years, 165 ± 7 cm, 66.5 ± 11 kg, 24.2 ± 2.8 kg/m^2^).

### 2.3. Testing Protocol

**Figure 2:**
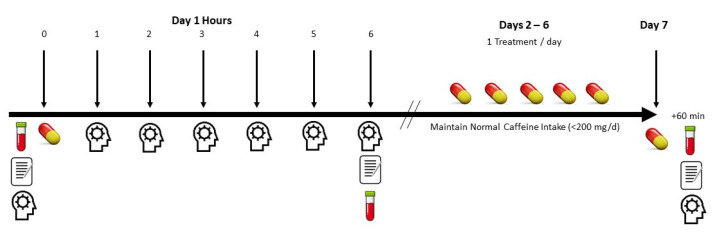
Overview of study protocol.

Figure 2 presents a study experiment timeline. Eligible volunteers were informed about the study protocol and signed informed consent statements. Participants then completed medical history forms and height, weight, heart rate, and blood pressure determined. A research assistant then described how to complete food and fluid intake records. Participants were provided a list of caffeine containing beverages and foods to avoid prior to each study session. Volunteers practiced each cognitive function test three times to establish test-retest reliability. Before testing sessions, participants were instructed to record food and fluid intake for 4-days. This record was used to replicate food and fluid intake prior to study sessions. Participants were also instructed to limit caffeine intake to less than 200 mg/d and refrain from consuming other stimulants not typically consumed in their habitual diet for 48 h prior to testing. Volunteers also observed an 8-h fast prior to testing sessions. Upon reporting to the lab, participants donated a fasting blood sample, completed a side-effects questionnaire, and performed pre-supplementation cognitive function tests. Each battery of cognitive function tests lasted about 35–40 min. Participants then ingested the assigned treatment with about 8 ounces of water. Participants completed the battery of cognitive function tests after 1, 2, 3, 4, 5, and 6 h. On days 2–6 of each treatment experiment, volunteers consumed one dose per day of the assigned treatment. On the 7th day, participants reported to the lab and ingested a final dose of the treatment. One hour later, participants donated fasting blood, completed a side effects survey, and performed the battery of cognitive function tests. Thus, this design allowed for acute and 7-day assessment of each treatment. Most participants observed a 7 to 14 day washout period between experiments. However, due to a university mandated 3-month suspension of research due to COVID during the study, several participants had a longer washout period between testing sessions. Subsequent treatments were administered in a crossover and counterbalanced manner. Participants replicated their 4-day diet, dietary restrictions, and 8-h fast as noted above before repeating each study session while consuming remaining treatments.

### 2.4. Supplementation Protocol

Supplements were administered in a randomized, double blind, crossover, and counterbalanced manner. Treatments included 200 mg of a wheat flour placebo (PLA, Shandong Bailong Chuangyuan Bio-tec Co. Ltd., Dezhou, China), 50 mg of PXN (PXN, ENFINITY™, Ingenious Ingredients, L.P., Lewisville, TX, USA) + 150 mg PLA (PXN 50), 100 mg PXN + 100 mg PLA (PXN 100), or 200 mg PXN (PXN 200). Supplements were manufactured following good manufacturing processes, tested for purity, and PXN has self-affirmed Generally Recognized as Safe (GRAS) status for use in food and beverages. Supplements were packaged in similar sized and colored capsules and placed in generically labeled bottles for double blind administration.

## 3. Procedures

### 3.1. Demographics

Participants’ height and weight were assessed using a self-calibrating (±0.02 kg) digital scale (Health-O-Meter Professional 500KL, Pelstar LLC, Alsip, IL, USA). Resting heart rate and blood pressure were obtained after sitting still for approximately 6-min. Heart rate was assessed via palpitation of the radial artery and blood pressure was measured by oscillation of the brachial artery using a mercurial sphygmomanometer [26].

### 3.2. Diet Control

In order to provide some dietary control prior to each study session, participants recorded all food and energy containing beverages for 4-days prior to the first testing session using the 2021 MyFitnessPal Calorie Counter phone application (MyFitnessPal, Inc., Baltimore, MD, USA) or written food logs. Participants replicated this diet prior to each treatment testing session. Food records were entered by research assistants and verified for consistency by one individual. Food records were analyzed using the Food Processor Nutrition Analysis Software, Version 11.4.412 (ESHA Nutrition Research, Salem, OR, USA) [27].

### 3.3. PEBL Cognitive and Executive Function Assessment

The Psychology Experiment Building Language (PEBL) cognitive function test battery (Version 2.1, http://pebl.sourceforge.net, accessed on 10 June 2019) was used to assess changes in cognitive function [28]. This included the Berg-Wisconsin Card Sorting Task test (BCST) [28,29,30,31,32], the Go/No-Go test (GNG) test [29,30,33], the Sternberg Task Test (STT) [29,30,34], and Psychomotor Vigilance Task Test (PVTT) [28,29,30,35,36] using methods previously described in detail [25]. Participants practiced the tests three times during familiarization sessions to establish test re-test reliability. Tests were performed in the same order with minimal delay between tests and took about 30–35 min to take. Participants relaxed between testing sessions.

### 3.4. Blood Colletion and Analysis

In order to assess the safety of 7 days of PXN ingestion at varying doses, we assessed standard whole blood and clinical blood panels. Approximately 15 mL of blood was collected from an antecubital vein in the forearm using standard phlebotomy procedures [37,38]. Blood was collected in 7.5 mL serum separator and 3.5 mL K2 EDTA BD Vacutainer^®^ tubes (Becton, Dickinson and Company, Franklin Lakes, NJ, USA). Serum separator tubes were left at room temperature for 15 min, centrifuged at 3500 × *g* for 10-min in a refrigerated (4 °C) Thermo Scientific Heraeus MegaFuge 40R Centrifuge (Thermo Electron North America LLC, West Palm Beach, FL, USA) [39]. Whole blood and serum samples were picked up daily and analyzed at Clinical Pathology Labs, Inc. (Austin, TX, USA, CLIA #45D0505003, CAP Accreditation #21525-01). Whole blood counts were analyzed using an automated multichannel hematology analyzer method with platelet count and 5-part differential determination. Serum samples were analyzed using Roche Cobas Gen 2 enzymatic/colorimetric analyzer (Roche Diagnostics International AG, Rotkreuz, Switzerland). Test-retest reliability of performing these assays ranged from 2% to 6%.

### 3.5. Side Effect Questionnaire

Participants rated the frequency and severity of dizziness, headache, tachycardia, heart skipping/palpitations, shortness of breath, nervousness, and blurred vision using a scale where 0 = none; 1 = 1–2/wk, 2 = 3–4/wk, 3 = 5–6/wk, 4 = 7–8/wk, and 5 = ≥ 9/wk and 0 = none, 1 = minimal, 2 = slight, 3 = moderate, 4 = severe, and 5 = very severe, respectively. Participants were also asked to report any other side effects experienced. Test re-test reliability in answering these questions in our lab have yielded mean coefficient of variation (CV) in the range of 1.2–2.6 and mean the intraclass correlation coefficient (ICC) range of 0.59–0.88 [40].

### 3.6. Statistical Analysis

The IBM^®^ Version 28 SPSS^®^ statistical package (IBM Corp., Armonk, NY, USA) was used to analyze data. Adequate sample size was determined based on an expected improvement of 5% with a power of 85% in primary outcome cognitive function related variables observed in our prior study [25,41,42,43]. The n-size evaluated also had sufficient power to assess clinically significant side effects consistent with similarly designed studies conducted in our lab [41,42,43,44,45]. Participants were randomized to treatments using a balanced Latin Square designer program [46]. Data were analyzed using general linear models (GLM) with repeated measures multivariate and univariate analyses using body weight (kg) as a covariate. Since this was a crossover design and no significant Treatment × Time × Sex interactions were observed, only treatment and time data are reported. A Mauchly’s test was used to assess sphericity. Normality was assessed using skewness and kurtosis statistics. Wilks’ Lambda distribution multivariate and Greenhouse-Geisser univariate correction tests were used to assess Time and Treatment × Time effects. Data were considered statistically significant when the probability of type I error (α-level) was 0.05 or less. Statistical tendencies noted when *p*-levels ranged between *p* > 0.05 to *p* < 0.10. Fisher’s least significant difference post-hoc analysis was used to assess pairwise differences. Clinical significance of findings was evaluated by calculating mean changes from baseline with 95% confidence intervals (CI). Mean changes with 95% CI’s completely above or below baseline were considered significantly different [47]. Sidak adjusted pairwise comparisons were performed to assess changes from baseline and among treatments. Data are presented as means ± standard deviations (SD) or mean mean changes from baseline (mean change ± SD (LL, UL)). Effect size was assessed using Partial Eta squared ($\eta_{p}^{2}$) values, where 0.01 represented a small effect, 0.06 represented a medium effect, and 0.14 represented a large effect size [48].

## 4. Results

### 4.1. Demographic Data

Twelve participants (9 males and 3 females) completed this study (see Table S1). Collectively, participants were 22.8 ± 4 years, 165 ± 7 cm, and 66.5 ± 11 kg with a body mass index (BMI) of 24.2 ± 3 kg/m^2^. Participants had normal resting heart rate (74.2 ± 10 bpm), systolic blood pressure (113.2 ± 10 mmHg), and a diastolic blood pressure (70.2 ± 4 mmHg). Since significant sex differences were seen in height and weight, body weight was used as a covariate in statistical analyses.

### 4.2. PEBL Cognitive Function Assessment

#### 4.2.1. Berg-Wisconsin Card Sorting Task Test

**Figure 3:**
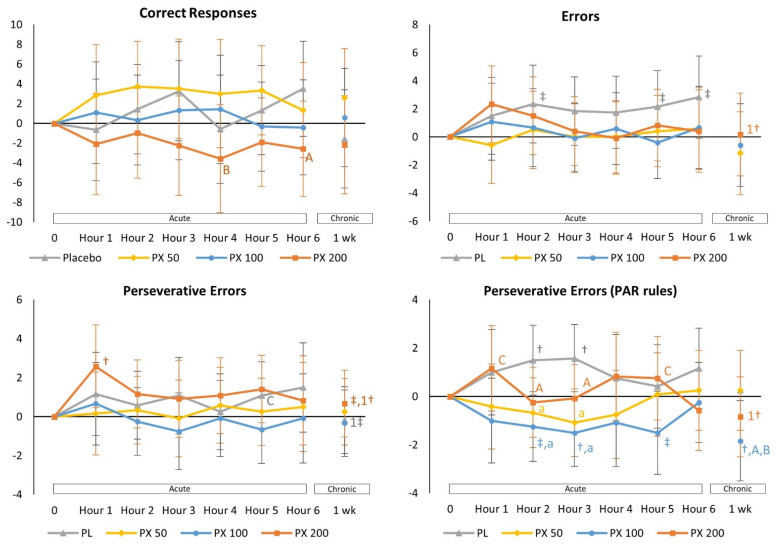
Mean changes with 95% confidence interval data from baseline in Berg-Wisconsin Card Sorting Test. † = *p* < 0.05 (‡ = *p* > 0.05 to *p* < 0.10) from baseline. 1† = *p* < 0.05 from Day1–1 h value. Treatment differences are denoted as from PLA: a = *p* < 0.05 (A = *p* > 0.05 to *p* < 0.10); PXN 50: b = *p* < 0.05 (B = *p* > 0.05 to *p* < 0.10); PXN 100: c = *p* < 0.05 (C = *p* > 0.05 to *p* < 0.10); and PXN 200: d = *p* < 0.05 (D = *p* > 0.05 to *p* < 0.10).

The BCST test involves sorting cards by matching colors and/or designs [28,29,30] and is used to assess accuracy, reaction time, thought, reasoning, learning, executive control, attention shifting, and impulsiveness [31,32]. GLM analysis of BCST results (see Table S2) revealed no significant overall or univariate treatment × time interaction effects in BCST related variables (i.e., Correct Responses, Errors, Perseverative Errors, or Perseverative Errors (PAR rules)). There was evidence (Figure 3) that the number of errors increased over time in the PLA treatment while being better maintained with PXN ingestion. This can clearly be seen in the perseverative errors using PAR rules analysis where errors increased over time with PLA treatment but were significantly lower than PLA in several of the PXN treatments after 2, 3, and 6 h. In this analysis, acute ingestion 100 mg of PXN appeared to be more advantageous compared to PLA treatment. Additionally, 7-days of PXN 100 ingestion sustained improved of Perseverative Errors (PAR Rules) and tended to be better than PLA and PXN 50 treatments. Total Errors and Perseverative Errors after 7-days were also significantly lower than Day 1–1 h values with PX200 treatment. Results provide some evidence that PXN 100 ingestion improved reaction time and accuracy in some BCST related variables.

#### 4.2.2. Go/No-Go Task Test

**Figure 4:**
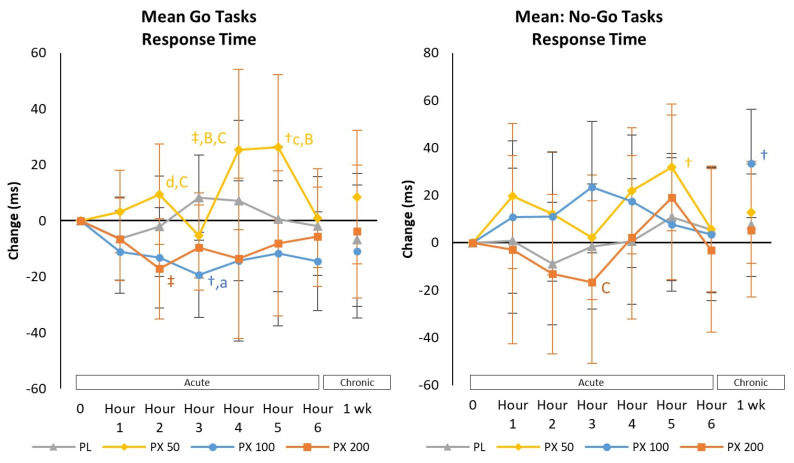
Mean changes from baseline with 95% confidence intervals in Go and No-Go task response times. † = *p* < 0.05 (‡ = *p* > 0.05 to *p* < 0.10) from baseline. 1† = *p* < 0.05 from Day 1–1 h value. Treatment differences are denoted as from PLA: a = *p* < 0.05 (A = *p* > 0.05 to *p* < 0.10); PXN 50: b = *p* < 0.05 (B = *p* > 0.05 to *p* < 0.10); PXN 100: c = *p* < 0.05 (C = *p* > 0.05 to *p* < 0.10); and PXN 200: d = *p* < 0.05 (D = *p* > 0.05 to *p* < 0.10).

The Go/No-Go test (GNG) was administered to assess sustained attention, response control in responding to visual stimuli, and impulsiveness [29,30,33]. As shown in Table S3, no significant overall or treatment × time effects were observed among GNG related variables. However, moderate effect sizes were observed suggesting an effect may be observed with more participants and/or greater consistency in responses. Assessment of mean changes from baseline revealed evidence that mean Go response time decreased over time in the PX 100 and PX200 and were significantly faster in the PX 100 treatment compared to PLA after 3 h (Figure 4). Go mean response time values in the PXN 100 and PXN 200 treatments were also generally better than responses observed in the PXN 50 treatment after 4 and 5 h of ingestion. No significant differences were observed among treatments in No-Go response time although the PXN 200 treatment tended to be faster than PXN 100 at 3-h. No significant differences were observed among treatments after 7 days of supplementation although response time was higher than baseline in the PX 100 treatment. These findings provide some evidence that acute ingestion of 100 mg and 200 mg of PXN help sustains attention and response control in responding to visual stimuli that require positive (Go) or inhibitory (No-Go) decision making skills.

#### 4.2.3. Sternberg Task Test

**Figure 5:**
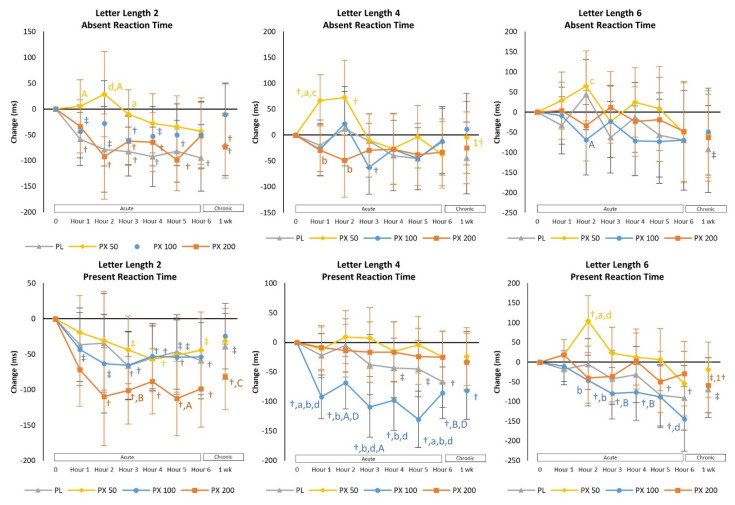
Sternberg Task Test mean (±95% Confidence Intervals) changes in Absent and Present Reaction Times. † = *p* < 0.05 (‡ = *p* > 0.05 to *p* < 0.10) from baseline. 1† = *p* < 0.05 from Day 1–1 h value. Treatment differences are denoted as from PLA: a = *p* < 0.05 (A = *p* > 0.05 to *p* < 0.10); PXN 50: b = *p* < 0.05 (B = *p* > 0.05 to *p* < 0.10); PXN 100: c = *p* < 0.05 (C = *p* > 0.05 to *p* < 0.10); and PXN 200: d = *p* < 0.05 (D = *p* > 0.05 to *p* < 0.10).

The STT test assess memory and cognitive control processes using reaction time and accuracy [29,30,34]. Although no significant overall or univariate interaction effects were observed (see Table S4), a treatment × time trend (*p* = 0.061) with a moderate to large effect size (*η_p_*^2^ = 0.103) was observed among Letter Length 4: Present Reaction Time responses. Additionally, some changes were seen within treatments over time. Analysis of mean changes from baseline in STT variables revealed evidence of improved Letter Length 2 Present Reaction Time in the PX 200 treatment as well as Letter Length 4 Present Reaction Time with PX 100 treatment compared to PLA (see Figure 5). There was also evidence that the improvement in Present Reaction Times generally improved during the first two hours after ingestion and were sustained for the remainder of the 6-h assessment period. After 7 days of supplementation, Letter Length 4 Absent Reaction Time values with PXN50 treatment and Letter Length 6 Present Reaction Time with PXN200 treatment were also significantly faster than Day 1–1 h values. These findings provide evidence that 100 mg and 200 mg of PXN ingestion improved measures of working memory involving cognitive control processes as the complexity of letter length tasks increased.

#### 4.2.4. Psychomotor Vigilance Task Test

**Figure 6:**
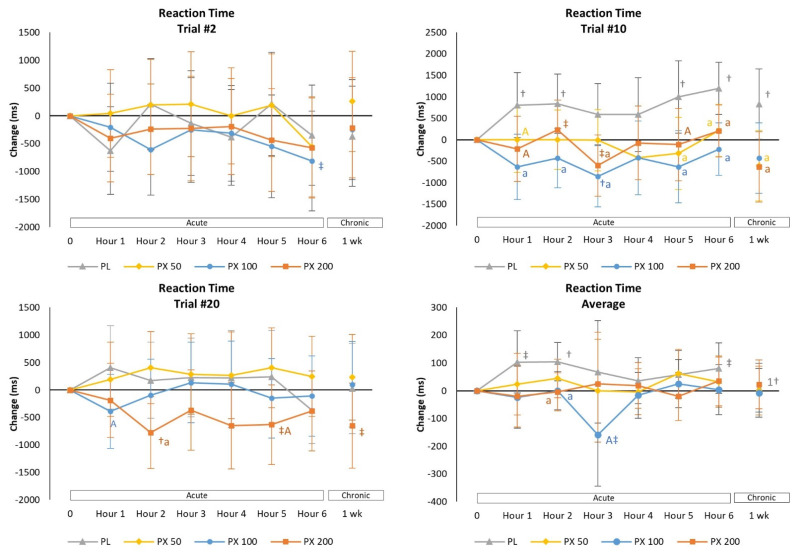
Mean changes with 95% Confidence Intervals in Psychomotor Vigilance Task Test data. † = *p* < 0.05 (‡ = *p* > 0.05 to *p* < 0.10) from baseline. 1† = *p* < 0.05 from Day 1–1 h value. Treatment differences are denoted as from PLA: a = *p* < 0.05 (A = *p* > 0.05 to *p* < 0.10); PXN 50: b = *p* < 0.05 (B = *p* > 0.05 to *p* < 0.10); PXN 100: c = *p* < 0.05 (C = *p* > 0.05 to *p* < 0.10); and PXN 200: d = *p* < 0.05 (D = *p* > 0.05 to *p* < 0.10.

The PVTT assesses sustained attention reaction times and sleepiness as determined by the number of lapses in attention [28,29,30,35,36]. Results in the present study revealed no significant overall or univariate interaction effects among treatments in PVTT results (see Table S5). However, moderate effect sizes were observed in some PVTT variables and with pairwise comparisons indicating some differences from baseline as well as over time suggesting that reaction times increased while improving over time with PLA treatment while being maintained or decreasing with PXN treatment. Analysis of mean changes from baseline revealed that there was a tendency for reaction time during Trial #2 to decrease at hour 6 in the PXN 100 treatment while no differences were observed among treatments (Figure 6). Reaction time in Trial #10 significantly increased over time with PLA while improving with PXN 100 and being significantly faster than PLA with PXN 50, PXN 100 and PXN 200 treatments at various points in time. Reaction time was also significantly improved in Trial #20 with PXN 200 treatment while PXN generally maintained average reaction time with PXN treatment while significantly increasing over time with PLA treatment. These findings suggest that PXN treatment helped sustained attention over time.

### 4.3. Safety Assessment

Tables S6–S9 present cell blood count and comprehensive metabolic panel analysis performed on 0 h, 6 h, and 7-day blood samples. No significant overall or univariate interaction effects were observed in whole blood cell counts with the exception of monocyte percent changes that showed no consistent changes among treatments and all values remained within norms (Table S6). No significant overall or univariate interaction effects were observed in blood lipid panels (Table S7), or markers of liver function (Table S8), markers of renal function (Table S9). Reports of side effects were infrequent, of minimal severity, and not significantly different among treatments (Table S10). Additionally, no participant withdrew from this study due to any side effects. These findings suggest that up to 7 days of PXN ingestion (50 mg–200 mg) was well tolerated.

## 5. Discussion

The purpose of this study was to examine the acute and short-term dose-related effects of PXN ingestion on markers of cognition, executive function and psychomotor vigilance. Additionally, to determine if acute or short-term ingestion of PXN causes any stimulant related side effects and/or adversely affects general clinical chemistry markers of health. Present findings provide additional evidence that PXN may serve as an effective nootropic. In this regard, there was evidence from BCST assessment that PXN ingestion improved reaction time. The BCST is used to assess thought, reasoning, learning, executive control, attention shifting, and impulsiveness [31,32]. Results revealed that PXN ingestion decreased the number of errors over time compared to PLA responses. This was most evident in decreasing the number of perseverative errors. Perseverative errors occur when the participant continues with the same response strategy following a rule switch or fails to inhibit a prepotent or dominate response [49]. These findings provide additional evidence that PXN ingestion can help sustain attention and improve accuracy over time. The Go/No-Go test was used to assess the ability to sustain attention, control responses to visual stimulation, reaction time, and accuracy [29,30,33]. We previously reported that acute ingestion of 200 mg of PXN decreased mean response time to Go Tasks while response time increased over time with PLA treatment [25]. We observed a similar response to 100 mg and 200 mg dosages in the present study, but not 50 mg dosage. These findings suggest that PXN ingestion can help sustain attention and response control to visual stimuli that require positive or inhibitory decision-making skills.

The Sternberg Task Test assesses reaction time, accuracy, and memory of tasks of increasing complexity [34,50,51,52]. In our initial study, we found ingestion of 200 mg of PXN improved reaction time more consistently than the PLA in shorter (2-letter) and longer (6-letter) memory challenges [25]. There was also evidence that reaction time improved more consistently over time as the number of trials progressed. In the present study, 200 mg of paraxanthine improved 2 letter length present reaction time to a greater degree while 100 mg of paraxanthine had greater effects on 4 letter and 6 letter length present reaction time. These findings provide additional evidence that PXN improved the ability to store and retrieve random information from memory of increasing complexity and helped sustain attention. Finally, participants performed the PVTT which assesses sustained attention reaction times to visual stimuli [28,35,36]. In our initial study [25], 200 mg of PXN better maintained average reaction time after 3-h. Results of the current study revealed that ingestion of 50 mg, 100 mg, and 200 mg of PXN improved reaction times at Trial #10 in comparison to the PLA treatment with the 200 mg dosage improved to a greater degree after Trial #20. However, ingestion of 100 mg and 200 mg of paraxanthine better maintained average reaction time while increasing in the placebo group. Results provide further evidence that PXN may help sustain attention over time.

Although participants in our initial study did not report any side effects [25], given that some individuals who consume caffeine and/or other stimulants experience unwanted side effects [2,53,54], we wanted to do additional safety evaluations. This included assessing the effects of acute and 7-days of paraxanthine supplementation (50 mg, 100 mg, and 200 mg) on clinical chemistry panels. Moreover, we administered a stimulant sensitivity questionnaire that asked participants to rate the frequency and/or severity of a number of common side effects associated with stimulant ingestion. This analysis revealed that ingestion of up to 200 mg of paraxanthine did not significantly affect cell blood counts, lipid profiles, markers of liver function, markers of renal function, or result in a changesin risk profile categories. Likewise, no significant differences among treatments were observed in the frequency or severity of dizziness, headache, tachycardia, heart palpitations, shortness of breath, nervousness, or blurred vision. It appeared that paraxanthine was well tolerated.

## 6. Conclusions

The strength of this study is that it provided the first known dose response study assessing the effects of PXN ingestion on several cognitive function tests in healthy volunteers. Additionally, it provided an initial assessment of the short-term effects of PXN supplementation on cognitive function and side effects. The study is limited by the sample size, tests performed, and assessment of healthy younger adults. It is also possible that individual variability may have influenced results. Nevertheless, present findings confirm our previous results [25] and support contentions that PXN may have nootropic properties in doses as low as 50 mg. There was also evidence that repeated daily ingestion sustained some of these improvements and/or were improved to a greater degree than after one hour of ingestion on Day 1. These findings also provide some preliminary evidence that there is no apparent habituation with up to 7 days of repeated paraxanthine ingestion at these dosages. Given that low-caffeine consumers have been reported to develop tolerance to chronic low dose ingestion of caffeine [55], it may be of interest to compare tolerance and habituation responses to caffeine and PXN to determine if PXN may offer long-term efficacy without habituation. Additional research should also evaluate whether PXN ingestion at rest and/or prior to exercise has similar effects as caffeine with less side effects. Results of our initial study and this more comprehensive dose efficacy and safety study are promising.

## Data Availability

Data and/or statistical analyses are available upon request on a case-by-case basis for non-commercial scientific inquiry and/or educational use as long as IRB restrictions and research agreement terms are not violated.

## Supplementary Materials

The following are available online at https://www.mdpi.com/article/10.3390/nu13124478/s1, Table S1: Demographic data, Table S2: Berg-Wisconsin Card Sorting Task Test results, Table S3: Go/No-Go Task Test results, Table S4: Sternberg Task Test results, Table S5: Psychomotor Vigilance Task Test results, Table S6: Whole Blood Cell Count analysis results, Table S7: Blood Lipid Profile results, Table S8: Markers of Liver Function results, Table S9: Serum Renal Function analysis results, Table S10: Frequency and Severity of Side Effects.
